# Healthy lifestyle behaviour among Ghanaian adults in the phase of a health policy change

**DOI:** 10.1186/1744-8603-7-7

**Published:** 2011-04-07

**Authors:** Henry A Tagoe, Fidelia AA Dake

**Affiliations:** 1 Regional Institute for Population Studies, University of Ghana, P.O. Box LG 96, Legon, Accra, Ghana

## Abstract

**Background:**

Many countries have adopted health policies that are targeted at reducing the risk factors for chronic non-communicable diseases. These policies promote a healthy population by encouraging people to adopt healthy lifestyle behaviours. This paper examines healthy lifestyle behaviour among Ghanaian adults by comparing behaviours before and after the introduction of a national health policy. The paper also explores the socio-economic and demographic factors associated with healthy lifestyle behaviour.

**Method:**

Descriptive, bivariate and multivariate regression techniques were employed on two nationally representative surveys (2003 World Health Survey (Ghana) and 2008 Ghana Demographic and Health Survey) to arrive at the results.

**Results:**

While the prevalence of some negative lifestyle behaviours like smoking has reduced others like alcohol consumption has increased. Relatively fewer people adhered to consuming the recommended amount of fruit and vegetable servings per day in 2008 compared to 2003. While more females (7.0%) exhibited healthier lifestyles, more males (9.0%) exhibited risky lifestyle behaviours after the introduction of the policy.

**Conclusion:**

The improvement in healthy lifestyle behaviours among female adult Ghanaians will help promote healthy living and potentially lead to a reduction in the prevalence of obesity among Ghanaian women. The increase in risky lifestyle behaviour among adult male Ghanaians even after the introduction of the health policy could lead to an increase in the risk of non-communicable diseases among men and the resultant burden of disease on them and their families will push more people into poverty.

## Background

The overall health of individuals is impacted by lifestyle behaviours including healthy diets, physical activity, smoking and alcohol consumption. Unhealthy lifestyle behaviours particularly poor dietary practices, physical inactivity and smoking are major risk factors for conditions like overweight, obesity and chronic non-communicable diseases [1-3]. Research in Ghana indicates that the prevalence of obesity is increasing especially among women [4]. The rising prevalence of obesity in Ghana is worrying because epidemiological studies have consistently shown an increased risk of morbidity, disability and mortality with obesity [5]. Findings from a study using data from a nationally representative sample survey (World Health Survey 2003) conducted in Ghana revealed that about 18% of the respondents had been diagnosed with one or more chronic non-communicable disease(s) with 45% of them currently receiving treatment (Tagoe, Household burden of chronic disease in Ghana, Unpublished). Health reports show that the prevalence of lifestyle diseases (chronic non-communicable diseases) such as stroke, hypertension, type 2 diabetes, and other cardiovascular diseases are on the increase and are now among the top ten in-patient cause of death in Ghana [6].

Urbanisation, globalisation and nutritional transition are major drivers of unhealthy lifestyle behaviours in developing countries [7-9]. Rapid urbanisation and globalisation is accompanied by behavioural change which exposes many individuals to the risk of chronic non-communicable diseases and mortality. Fast paced economic transition has also resulted in reduced physical activity levels, decreased hours of rest and increasing levels of stress [8,9].

The progressive increase in the burden of chronic non-communicable diseases has been attributed to several factors including longer average lifespan and risky lifestyle behaviours [10]. Tobacco use, physical inactivity and diets high in saturated fat and salts constitute risk for conditions such as cardiovascular diseases, high blood pressure and elevated serum cholesterol levels [11-13]. While factors such age, sex and genetic susceptibility are non-modifiable many of the risks associated with chronic diseases are modifiable. Such modifiable risks include behavioural factors (e.g. diet, physical inactivity, tobacco use, alcohol consumption), medical conditions (e.g. dyslipidemia, hypertension, overweight, hyperinsulinaemia) and societal factors including include a complex mixture of interacting socioeconomic, cultural and environmental factors [14,15]. Estimates by the World Health Organisation suggest that up to 80% of premature deaths from heart disease, stroke and diabetes can be averted with known behavioural and pharmaceutical interventions [16]. According to the Archives of Internal Medicine (1997) [17], the prevention of hypertension by means of dietary salt reduction and weight loss over a short term has been successfully accomplished in clinical trials. It has also been identified that diets high in fruits, vegetables and low-fat dairy products are extremely effective in lowering blood pressure [18].

From the foregoing, it is evident that the increase in the incidence and prevalence of non-communicable diseases are linked to risky healthy lifestyle behaviours [19]. Thus populations that exhibit risky lifestyle behaviours are also at risk of having a double burden of disease and poverty as is currently seen in developing also referred to as the Global South. In an effort to curb this pattern of disease and poverty many countries in the Global South have initiated and implemented health policies and intervention programs to help improve the health of their populations. Most of these interventions have, however, not yielded the expected results due to implementation problems and non-adherence to recommended healthy lifestyle behaviours.

The Ministry of Health (MOH) in Ghana as part of its effort to reduce the incidence of preventable diseases and to promote regenerative health in the country adopted the concept of "Regenerative Health and Nutrition (RHN)". The main objective of the program is to promote healthy lifestyles, dietary practices and mother and child care practices that would help eliminate the many diseases that impact on the health and well-being of Ghanaians. The concept of regenerative health and nutrition was adopted by the MOH from Dimona, Israel, where a community of more than 3,000 African Hebrews have lived for over 40 years without any recorded deaths among the people during this period [20]. Due to healthy lifestyle behaviours (including the adoption of vegan diets), the African Hebrews have been able to eliminate hypertension, diabetes, cancer and other chronic non-communicable diseases from their community [21].

The program covers three main modules; (a) mother and child care (b) healthy lifestyle and (c) regenerative nutrition [22]. Key interventions under the program are geared towards; healthy diet (increasing consumption of fruits and vegetables, drinking more water, reducing the intake of meat, salt and saturated oils/fats, reducing or eliminating smoking and alcohol intake); exercise (increasing daily physical activity including cardiovascular exercise); rest (adopting regular relaxation practices to minimise physical and emotional stress) and environmental sanitation (maintaining personal and environmental cleanliness and advocating for portable water use). Under these interventions it is recommended that individuals consume five servings each of fruits and vegetables and also drink eight glasses of water a day. Living in a clean environment is encouraged and smoking and alcohol consumption are to be avoided.

The Ghana Regenerative Health and Nutrition Program was adopted in 2005 and piloted in 2006. The initial pilot involved ten districts across seven administrative regions. As part of the pilot program about 700 change agents and 5000 advocates were trained [23]. Change agents and advocates of the program are members of the community who are trained in the principles and practices of RHN and they in turn educate their community members [22]. The program has trained over 50,000 change agents and advocates throughout the country over the four year period (2006 to 2010) [24]. Mass communication through the use of both print and electronic media serves as a means of reaching the population with the messages of the program.

In this paper the authors compare the prevalence of unhealthy lifestyle behaviours among Ghanaian adults before and after the adoption of the regenerative health and nutrition program with a focus on behaviours including fruit and vegetable consumption, physical activity, smoking and alcohol consumption. This paper also assesses the trend and the socio-economic and demographic determinants of healthy lifestyle behaviours among Ghanaian adults prior to and after the introduction of this policy. The paper also highlights the implication of unhealthy lifestyle behaviour on morbidity and mortality in the country. The authors hope this paper will generate a new research agenda and also bring to bear the health challenges risky lifestyle behaviours pose to developing countries.

## Methodology

### Data

This paper combines data from two nationally representative population surveys conducted in Ghana - the World Health Survey (WHS) conducted under the WHO in 2003 and the Ghana Demographic and Health Survey (GDHS) conducted in 2008. The 2003 WHS targeted the *de facto *population aged 18 years and older. Households were selected using a random stratified sampling procedure. One individual per household was selected through a random selection procedure using the Kish table method. There was a known non-zero selection probability for any individual included in the study for the purposes of extrapolating the data to the whole population and the sampling strategy was without replacement. A total of 5,662 households were sampled out of which 4,121 were interviewed while in the case of individuals, 4,005 were sampled and 3,873 were interviewed. The 2008 GDHS, which is the fifth round in the series collected demographic, socio-economic and health information on men and women in their reproductive ages (females; 15-49 years and males; 15-59 years) and also on children under the age of five years. The sampling technique for the 2008 GDHS involved a two-stage stratified probability design. The first stage involved selecting clusters from an updated master sampling frame constructed from the 2000 Ghana Population and Housing Census. A total of 412 clusters were selected using systematic sampling with probability proportional to size. The second stage of selection involved a systematic sampling of 30 of the households listed in each cluster. Adult respondents in the 2008 GDHS included 4,916 females and 4,568 males in their reproductive ages. Both surveys collected information on healthy lifestyle behaviours including physical activity, fruit and vegetable consumption and also on smoking and alcohol consumption.

### Variables

#### Dependent variable

An index of healthy lifestyle behaviour computed based on the health related behaviours was used as the dependent variable. The components of the index were (i) physical activity, i.e. whether respondents engaged in any vigorous physical activity that lasted more than 10 minutes and the number of days respondents engaged in such activity in the last seven days preceding the survey. (ii) Smoking - this was a multiple response variable which was computed based on whether respondents engaged in at least one of the following: smoked or used any other nicotine containing substance in the last seven days preceding the survey. (iii) Alcohol consumption - whether or not respondents consumed at least one standard measure (Standard measure of alcohol is a net alcohol content of between 8-13 g of ethanol [1 standard bottle of regular beer(285 ml), 1 single measure of spirit (30 ml), 1 medium size glass of wine (120 ml) and 1 measure of aperitif (60 ml)] (WHS 2002)) of alcoholic beverage in the last seven days preceding the survey (iv) Fruits and vegetables - the amount of fruit and vegetable servings respondents consumed on average in a typical day.

A factor analysis using the principal component method was used to compute the index of healthy lifestyle behaviour. For two of the healthy lifestyle behaviours considered (smoking and alcohol consumption), a score of zero was assigned to a response indicating negative behaviour. Example, if a respondent reported smoking in the last seven days preceding the survey, zero was assigned if not one was assigned. In a similar manner, zero was assigned if a respondent reported consuming alcohol in the last seven days and one was assigned if no alcohol consumption was reported. The amount of fruits and vegetables consumed was reported as a count of the number of servings consumed while vigorous physical activity was reported as the number of days respondents engaged in vigorous physical activity that lasted for at least 10 minutes in the last 7 days preceding the survey. In the multivariate model the index was treated as a continuous linear variable. At the bivariate stage of analysis, the dependent variable was categorized into three equal parts based on the distribution of the computed index. The lowest 33.33% was categorised as "high risk" healthy lifestyle behaviour. The second 33.33% was categorised as "moderate risk" healthy lifestyle behaviour while the upper 33.33% was categorised as "low risk" healthy lifestyle behaviour. At the univariate level, the individual healthy lifestyle behaviours were categorised based on the recommendations for that behaviour. Amount of fruit and vegetable servings consumed per day on a typical day during the last week preceding the survey were categorised into three; none (0 servings), below the recommended amount (1-4 servings) and recommended amount (5 or more servings). Number of vigorous physical activity days during the last 7 days preceding the survey was also categorised into none (no vigorous physical activity in the last 7 days), 1-6 days and all 7 days of the week. With regards to smoking and alcohol consumption the respondents were grouped into the percentage that reported smoking and the percentage that reported consuming alcohol.

### Independent variables

The socio-economic and demographic characteristics of the respondents were used as independent variables and they included age, type of place of residence, marital status, highest level of educational attainment, type of occupation and household income quintile. There were differences in the age brackets for the different surveys, while the WHS focused on adults aged 18 years and older, the DHS concentrated on adults in their reproductive ages; females 15-49 years and males 15-59 years. To address the differences in age brackets the intersection of age in both datasets was used for the analysis, thus limiting respondents to adults aged 18-49 years. Also, all other measures of variables used were categorized to allow for cross survey comparison. Age had four categories of 18-19, 20-29, 30-39 and 40-49 years. Respondents were classified by sex; male or female and by type of place of residence; rural or urban. With regards to marital status respondents were classified as never married, currently married/cohabiting or formerly married. Based on their highest level of educational attainment respondents were put into categories of those with no formal education and those who had primary, secondary or higher than secondary level education. Occupational categories included those not working, professional workers including (technical, managerial and clerical workers), those in the sales/service and agriculture/fishery sectors and those engaged in elementary work including plant/machine operators.

### Methods of analysis

Statistical analysis carried out in this study employed descriptive, bivariate and multivariate regression techniques. Lifestyle behaviours, socio-economic and demographic characteristics of the respondents were explored using descriptive statistics. Bivariate analysis was used to assess the association between healthy lifestyle behaviour and the socio-economic and demographic characteristics of the respondents. To investigate the relationship between the individual demographic and socio-economic status variables (age, educational attainment, marital status, occupation, type of place of residence, and household income quintile) and healthy lifestyle behaviour we used a multivariate linear regression technique.

## Results

### Prevalence of risky lifestyle behaviours

Fewer males and females reported smoking in 2008 compared to 2003. In contrast, more males and females reported consuming alcohol in 2008 compared to 2003 (Table 1). The proportion of respondents who did not consume any servings of fruits increased by at least 10 percentage points while the proportion that consumed 5 or more servings of fruits decreased substantially among males and females alike. Similarly, the proportion of respondents who reported consuming a minimum of five servings of vegetables a day decreased by at least 6 percentage points. About 9 in 10 of the respondents reported consuming between 1 and 4 servings of vegetables before the introduction of the program and this pattern remained the same after the introduction of the program (Table 1). More than half of the female respondents did not engage in any form of vigorous physical activity before and after the introduction of the policy. However, among males, the proportion that did not engage in vigorous physical activity decreased by 8 percent. There was a marginal increase in the percentage of respondents who engaged in vigorous physical activity after the introduction of the program.

**Table 1:** Prevalence of risky lifestyle behaviours among Ghanaian adults, 2003 and 2008

	2003		2008	
	
Lifestyle behaviour	Females (%)	Males (%)	Females (%)	Males (%)
**Smoking**^**†**^	19 (1.3)	158 (12.4)	23 (0.5)	315 (9.0)
**Alcohol**^**‡**^	205 (13.5)	338 (30.4)	661 (15.4)	1,147 (32.7)
**Number of servings of fruit per day**				
None (0)	59 (3.9)	82 (6.4)	669 (15.5)	595 (17.0)
1-4 servings	1,052 (69.3)	810 (63.4)	3,516 (81.7)	2,875 (82.1)
5 or more servings	408 (26.9)	386 (30.2)	121 (2.8)	33 (0.9)
**Number of serving of vegetable per day**				
None (0)	30 (2.0)	48 (3.8)	215 (5.0)	262 (7.5)
1-4 servings	1,373 (90.4)	1,122 (87.8)	4,028 (93.5)	3,211 (91.7)
5 or more servings	116 (7.6)	108 (8.5)	63 (1.5)	30 (0.9)
**Vigorous physical activity (No. of days)**				
None	883 (58.2)	429 (33.6)	2,281 (53.0)	898 (25.6)
1-6	507 (33.4)	626 (49.0)	1,604 (37.3)	1,954 (55.8)
7	129 (8.5)	223 (17.4)	421 (9.8)	651 (18.6)
**Total N**	**1,519**	**1,278**	**4,306**	**3,503**

Source: Computed from the GWHS 2003 and GDHS 2008

^**† **^Respondents who reported smoking

^**‡ **^Respondents who reported consuming alcohol

### Healthy lifestyle behaviour

More females reported healthier lifestyles after the introduction of the program whereas more males on the other hand reported living riskier lifestyles after the introduction of the program (Tables 2 and 3). More females in rural areas reported living healthier lifestyles after the program was introduced. Interestingly, there was a 13 percentage point increase in the percentage of rural male residents who exhibited risky lifestyle behaviours after the RHN program was introduced. Similarly, while more urban females reported living low risk lifestyles in 2008 compared to 2003 more urban males reported high risk lifestyles in 2008 compared to 2003. The proportion of females who reported living healthier lifestyles after the introduction of the program increased across all age groups. The situation was the reverse among males, more males reported living riskier lifestyles now (2008) than before (2003) and this cut across all age groups. More males with primary education exhibited riskier lifestyles after the introduction of the program. The proportion of female professional workers who exhibited low risk lifestyles after the introduction of the program was about twice the proportion that reported such lifestyles before the introduction of the program (Tables 2 and 3). More females in all income categories reported healthier lifestyles in 2008. However, among males, the percentage that reported living healthier lifestyles in 2008 decreased across all income categories except the richest (Table 3).

**Table 2:** Percentage distribution of respondents by demographic and socio-economic characteristics and healthy lifestyle behaviour (2003)

Socio-economic and demographic characteristics	Healthy lifestyle behaviour					
	
	Females			Males		
		
	High	Moderate	Low	High	Moderate	Low
**Type of place of residence**			***			***
Urban	43.9	35.7	20.4	33.4	34.8	31.8
Rural	32.3	38.0	29.7	18.6	33.4	48.0
**Age group**						
18-19	40.0	38.8	21.2	23.8	41.6	34.7
20-29	37.6	37.6	24.8	25.1	34.7	40.2
30-39	36.8	34.7	28.5	23.5	34.7	40.2
40-49	35.8	39.2	24.9	24.6	32.0	43.6
**Marital status**			**			***
Never married	46.2	32.1	21.7	30.0	35.4	34.6
Currently married/cohabiting	34.4	37.5	28.1	20.8	32.2	47.0
Formerly married	37.1	41.8	21.1	22.1	42.6	35.3
**Highest level of educational attainment**						***
No formal education	31.5	38.6	29.9	19.1	28.0	52.9
Primary	39.6	36.1	24.4	22.1	36.3	41.6
Secondary	41.5	36.8	21.7	38.5	33.6	28.0
Higher	34.8	43.5	21.7	44.8	27.6	27.6
**Main occupation**			***			***
Not working	44.0	37.7	18.3	29.2	38.6	32.2
Professional/managerial/clerical	53.0	31.6	15.4	46.3	32.9	20.8
Service and sales	41.0	37.0	22.0	30.7	39.2	30.2
Agricultural and fishery	25.7	39.2	35.1	13.6	31.1	55.3
Plant/machine operators and elementary work	47.1	31.8	21.0	30.4	30.4	39.1
**Income quintile**			***			***
Poorest	29.5	39.5	31.0	15.4	31.6	53.1
Poor	28.1	36.6	35.3	12.3	36.8	50.9
Middle	32.0	39.8	28.2	15.7	34.8	49.6
Rich	42.0	35.6	22.4	32.4	36.3	31.3
Richest	49.5	33.4	17.0	39.2	31.9	28.9
**Total**	**37.0**	**37.1**	**25.9**	**24.3**	**34.0**	**41.7**

High = Less healthy (more risky behaviours) Low = More healthy (less risky behaviours)

***P < 0.001 **P < 0.01 *P < 0.05 Total N (Females = 1519, Males = 1278)

Source: Generated from WHS-Ghana 2003

**Table 3:** Percentage distribution of respondents by demographic and socio-economic characteristics and healthy lifestyle behaviour (2008)

Socio-demographic and economic characteristics	Healthy lifestyle behaviour					
	
	Females			Males		
		
	High	Moderate	Low	High	Moderate	Low
**Type of place of residence**			***			***
Urban	38.4	34.1	27.6	35.7	36.0	28.3
Rural	30.1	32.7	37.2	31.5	30.6	37.9
**Age group**			**			**
18-19	31.9	37.7	30.4	40.5	32.2	27.3
20-29	35.5	34.2	30.4	34.5	32.8	32.7
30-39	33.9	32.1	34.1	31.9	33.9	34.2
40-49	31.4	31.5	37.1	29.5	32.0	38.5
**Marital status**			**			***
Never married	33.8	37.5	28.7	37.1	32.1	30.9
Currently married/cohabiting	33.5	32.5	34.1	30.9	32.9	36.2
Formerly married	35.7	29.7	34.5	30.1	40.4	29.5
**Highest level of educational attainment**			*			*
No formal education	33.1	31.5	35.5	38.4	28.5	33.1
Primary	33.9	30.9	35.2	32.7	31.3	36.1
Secondary	34.1	35.0	30.9	31.9	33.9	34.2
Higher	33.7	37.6	28.7	33.0	37.9	29.1
**Main occupation**			***			***
Not working	40.7	37.6	21.7	40.7	31.3	28.0
Professional/managerial/clerical	29.3	40.4	30.2	33.4	38.1	28.5
Service and sales	35.0	34.3	30.8	32.1	42.7	25.2
Agricultural and fishery	27.4	29.3	43.2	30.4	28.5	41.1
Plant/machine operators and elementary work	38.3	30.2	31.4	34.2	22.9	31.9
**Income quintile**			***			***
Poorest	29.6	30.5	39.9	35.8	29.0	35.3
Poor	28.4	33.1	38.6	26.4	27.9	45.6
Middle	33.0	32.8	34.2	32.3	33.0	34.8
Rich	39.0	32.5	28.5	34.5	35.4	30.2
Richest	38.6	37.9	23.4	36.5	39.0	30.2
**Total**	**33.8**	**33.3**	**32.9**	**33.3**	**32.9**	**33.8**

High = Less healthy (more risky behaviours) Low = More healthy (less risky behaviours)

***P < 0.001 **P < 0.01 *P < 0.05 Total N (Females = 4,306, Males = 3,503)

Source: Generated from GDHS, 2008

### Socio-economic and demographic correlates of healthy lifestyle behaviour

Controlling for the independent variables in a multivariate regression model revealed that certain socio-economic and demographic variables are associated with healthy lifestyle behaviour (Table 4). Residing in an urban area was generally associated with unhealthy lifestyle behaviour though the relationship was observed to be non-significant except among urban males in 2003. The results suggests that education was associated with negative behaviours before the introduction of RHN, however, in the era of the health policy (i.e. in 2008), having formal education was generally associated with living healthy with the chances of making healthy adjustments increasing with increasing level of education. Among males the chances of living healthy increased with increasing level of educational attainment from primary through to higher level of education whereas among females, secondary through higher level educational attainment was associated with living a healthier lifestyle. Females in all occupational categories showed prospects of living healthy after the introduction of RHN. Even though statistical significance was not achieved, being a professional female worker was associated with living unhealthy before the introduction of RHN. However, after the introduction of RHN female professional workers were significantly more likely to live healthier lifestyles. Being a male professional worker was significantly associated with living unhealthy before the introduction of RHN but after the introduction of the program being a male professional worker was associated with higher chances of living healthy even though this was not statistically significant. Agricultural workers continued to live healthy even though the chances of doing so reduced after the introduction of RHN. Being a female in the rich or richest income quintile was associated with a higher chance of living less healthy before RHN was introduced. Even though this relationship was not significant before the introduction of RHN it persisted even after RHN was introduced with the relationship showing statistical significance. Being a female in the middle income quintile was associated with living less healthy after RHN but the opposite was the case before the introduction of RHN. Being a male in the poor and middle quintiles was associated with living healthy before and after the introduction of RHN (Table 4).

**Table 4:** Socio-economic and demographic correlates of healthy lifestyle behaviour among Ghanaian adults (2003 and 2008)

Socio-demographic variables	2003 B (Std. Error)		2008 B (Std. Error)	
		
	Females	Males	Females	Males
**Constant**	1.489 (.374)***	2.454 (.456)***	1.827 (.076)***	1.686 (.094)***
**Type of place of residence**				
Rural^◙^				
Urban	-.136 (.178)	-.493 (.246)*	-.038 (.048)	-.025 (.056)
**Age**				
18-19^◙^				
20-29	.251 (.363)	.281 (.393)	-.052 (.067)	.035 (.074)
30-39	.176 (.388)	-.112 (.448)	.000 (.076)	.023 (.090)
40-49	.206 (.404)	-.398 (.475)	.043 (.080)	.068 (.096)
**Marital status**				
Never married^◙^				
Married/cohabiting	-.246 (.229)	.784 (.275)**	-.069 (.053)	.111 (.060)
Formerly married	-.489 (.295)	.040 (.487)	-.059 (.075)	-.009 (.109)
**Highest level of educational attainment**				
No formal education^◙^				
Primary education	-.088 (.167)	-.639 (.250)*	.089 (.053)	.318 (.074)***
Secondary education	-.063 (.322)	-.634 (.376)	.188 (.049)***	.426 (.065)***
Higher education	-.331 (.605)	-.570 (.523)	.252 (.112)*	.521 (.101)***
**Main occupation**				
Not working^◙^				
Professional	-.478 (.333)	-.864 (.409)*	.338 (.097)***	.045 (.080)
Sales/services	.116 (.242)	.145 (.353)	.281 (.054)***	.012 (.089)
Agriculture/fishery	.848 (.248)**	1.110 (321)**	.481 (.063)***	.216 (.076)**
Plant/machine operators and elementary work	.273 (.297)	.478 (.444)	.170 (.071)*	.049 (.076)
**Income quintile**				
Poorest^◙^				
Poor	.269 (.223)	.736 (.298)*	.003 (.056)	.336 (.066)***
Middle	.289 (.229)	.603 (.303)*	-.129 (.063)*	.176 (.077)*
Rich	-.165 (.242)	-.009 (.315)	-.207 (.070)**	. 121 (.085)
Richest	-.077 (268)	.352 (.366)	-.294 (.077)***	-.044 (.094)

◙ = Reference category ***P < 0.001 **P < 0.01 *P < 0.05

Total N 2003 (Females = 1,519, Males = 1,278) 2008 (Females = 4,306, Males = 3,503)

Source: Generated from WHS-Ghana 2003 and GDHS, 2008

## Discussion

This paper examined the trend in healthy lifestyle behaviour among Ghanaian adults in the phase of the "Regenerative Health and Nutrition" health policy. Our findings reveal an increase in risky lifestyle behaviour among males and a decrease in risky lifestyle behaviour among females after the RHN program was introduced. The results of this study also revealed that risky lifestyle behaviours are more common in urban areas compared to rural. This result buttresses the argument that urban areas in developing countries are increasing becoming unhealthy environments in terms of lifestyle behaviours compared to rural areas. This trend may be partly responsible for the higher prevalence of obesity and non-communicable diseases in urban areas of developing countries as reported by the World Health Organisation [25].

It was also found that prior to the introduction of the program, in 2003, Ghanaian adults who had some level of education were less likely to exhibit healthy lifestyle behaviours. In 2008, after the introduction of the program, a reversed trend between educational attainment and healthy lifestyle behaviour was observed. Ghanaian adults were more likely to live a healthier lifestyle with increasing levels of educational attainment. The significant decline in risky lifestyle behaviour among the highly educated and among professional workers in 2008 after the introduction of the regenerative health and nutrition health policy in Ghana brings to the fore issues of access to regenerative health and nutrition information and the financial ability to effect a lifestyle behaviour change. The relatively high income level of professional workers gives them the opportunity to access the appropriate nutrition in terms of fruits and vegetables recommended under the program. Having high education also means they are an audience who can be reached with the messages of the program and thus they are more likely to change their behaviour. Behaviour change among the highly educated and professional workers does not end there. It is also more likely to be sustained since people of such calibre are also able to integrate the changed behaviour into their everyday lives and this is because they have the financial means, the knowledge base and the autonomy to be able to do so [26].

Improvement in the economic conditions of people is an asset but can be a liability as well. As revealed by this study, increasing income levels is generally associated with living risky lifestyles. This is especially so because people tend to engage in luxurious lifestyles including unhealthy snacking, consumption of high fat diets and sedentary lifestyles as their economic condition improves. This is a common occurrence in developing countries because such luxurious lifestyles are deemed prestigious and are also seen as a sign of wealth [1]. Such lifestyle behaviours, however, are unhealthy and have implications for the incidence of non-communicable diseases and mortality in developing countries.

This study gives preliminary results and shows the changes in lifestyle behaviours immediately before and after the introduction of the regenerative health and nutrition policy in Ghana. While this study makes important contributions to this area of research the results are likely to be influenced by differences in survey design since data from two comparable but different surveys were used for the analysis. This notwithstanding, measures in both surveys are similar to each other and this allows for cross survey comparison. To effectively evaluate such a program it is important to continually monitor the program. The authors thus recommend that future rounds of the Ghana Demographic and Health Survey continue to collect data on the program. This will allow for continuous monitoring of the program while making data available for tracking changes over time.

## Conclusion

The findings of this study has implications for the health and economic well being of Ghanaians and also for the future of the regenerative health and nutrition program in Ghana. The decreasing prevalence of risky lifestyle behaviours among females will help promote healthy living among females and potentially lead to a reduction in the prevalence of obesity among females which would counter the recent rise in obesity levels among Ghanaian women. The increase in risky lifestyle behaviour among males in spite of the regenerative health and nutrition program could lead to an increased risk of non-communicable diseases among males. This will not only defeat the objective of the program but also lead to morbidity and mortality.

While efforts aimed at promoting healthy lifestyle among females should be sustained more efforts need to be channelled at men in getting them to live healthier lifestyles. There is also the need to pay more attention to urban areas. While is important to promote healthy lifestyles in urban areas, there is also a need to target barriers in the urban environment that does not support the adoption of healthy lifestyles. These findings provide the leverage for further assessment of the regenerative health and nutrition health initiative on healthy lifestyle behaviours and its influence on morbidity and mortality. Additional research should attempt to explain the changes in healthy lifestyle behaviour among men and women in opposite directions. Exploring methods of targeting messages of healthy lifestyle behaviour choices and ways of making such options financially possible will foster the adoption of the regenerative health and nutrition program in other countries in the Global South.

## Competing interests

The authors declare that they have no competing interests.

## Authors' contributions

HAT developed the conceptual approach and performed the statistical analysis. FAAD drafted and revised the manuscript. Both authors developed the study design and reviewed and approved the final manuscript.
